# miR‐106b‐5p promotes aggressive progression of hepatocellular carcinoma via targeting RUNX3

**DOI:** 10.1002/cam4.2511

**Published:** 2019-09-10

**Authors:** Hao Gu, Shensen Gu, Xinlong Zhang, Songjiang Zhang, Dongming Zhang, Junsheng Lin, Saiken Hasengbayi, Wei Han

**Affiliations:** ^1^ Digestive Vascular Surgery Center Xinjiang Medical University Xinjiang China

**Keywords:** cell viability, hepatocellular carcinoma, invasion, microRNA‐106b‐5p, overall survival, recurrence‐free survival

## Abstract

**Background and Objectives:**

The roles of microRNA(miR)‐106b‐5p in hepatocellular carcinoma (HCC) remain unclear. We aimed here to investigate the clinical significance of miR‐106b‐5p expression in HCC and its underlying mechanisms.

**Methods:**

Expression levels of miR‐106b‐5p in 108 HCC clinical samples by quantitative real‐time reverse transcription PCR. Associations of miR‐106b‐5p expression with various clinicopathological features and patients' prognosis were evaluated by Chi‐square test, Kaplan‐Meier, and Cox proportional regression analyses, respectively. The target gene of miR‐106b‐5p and their functions in HCC cells were investigated by luciferase reporter, CCK‐8, and Transwell Matrigel invasion assays.

**Results:**

miR‐106b‐5p expression was markedly higher in HCC tissues than in noncancerous adjacent liver tissues (*P* < .001). miR‐106b‐5p upregulation was significantly associated with advanced TNM stage (*P* = .02), short recurrence‐free (*P* = .005), and overall (*P* = .001) survivals. Importantly, miR‐106b‐5p expression was an independent predictor of poor prognosis (*P* < .05). RUNX3 was identified as a direct target gene of miR‐106b‐5p in HCC cells. Functionally, miR‐106b‐5p upregulation promoted the viability and invasion of HCC cells, while enforced RUNX3 expression reversed the oncogenic effects of miR‐106b‐5p overexpression.

**Conclusions:**

miR‐106b‐5p may serve as a potent prognostic marker for tumor recurrence and survival of HCC patients. miR‐106b‐5p may exert an oncogenic role in HCC via regulating its target gene RUNX3.

## Graphical abstract

This study revealed the overexpression of miR‐106b‐5p in HCC tissues, which was dramatically associated with advanced cancer progression and unfavorable patients' prognosis. Functionally, miR‐106b‐5p upregulation promoted the viability and invasion of HCC cells, at least in part, by inhibiting its target gene RUNX3

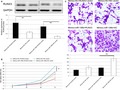

## 1 INTRODUCTION

Hepatocellular carcinoma (HCC) accounts for more than ninety percent of all liver cancers and may be the third leading cause of cancer mortality, with approximately 700 000 deaths in the world annually.^1^ Till now, surgery resection remains the main curative option for HCC. However, most HCC patients are diagnosed at advanced tumor stages, characterized by multifocal progression, lymph node metastasis, and portal vein invasion, almost seventy percent of HCC patients cannot adapt to the curative hepatectomy.^2^, ^3^ Due to early metastasis and high frequency of recurrence, the prognosis of most HCC patients is very poor. Growing evidence show that the development and progression of HCC may be a multistep process which may be involved by numerous genes, proteins, and noncoding RNAs.^4^, ^5^ Therefore, a comprehensive understanding of novel molecular markers and pathways that contribute to the progression and recurrence of HCC is necessary to develop more efficient and targeted therapies.

MicroRNAs (miRNAs) represent a cluster of short, endogenous, single‐strand and noncoding RNAs with approximately 19 to 25 nucleotides in length. miRNAs may regulate the corresponding target gene expression post‐transcriptionally in almost all aspects of normal physiological and pathological processes.^6^, ^7^, ^8^, ^9^ Recent studies have observed the altered expression of numerous miRNAs in HCC tissues and cells. The upregulated miRNAs often play oncogenic roles by negatively regulating tumor suppressor genes, whereas the downregulated miRNAs exert tumor‐suppressive functions via suppressing oncogenes.^10^, ^11^, ^12^ miR‐106b‐5p is transcribed from the miR‐106b～25 cluster located on chromosome 7.^13^ It belongs to the miR‐106b seed family which has been indicated to be closely associated with cell proliferation, cell cycle, and cell motility.^13^ Accumulating studies have reported that miR‐106b‐5p plays an important role in various cancers through diverse mechanisms.^14^, ^15^, ^16^, ^17^, ^18^, ^19^, ^20^ Particularly, Shi et al^21^ found that the expression of miR‐106b‐5p was higher in HCC tissues and cell lines than that in nontumor tissues and hepatocytes, respectively. The authors also indicated that miR‐106b‐5p upregulation promoted stem cell‐like properties of HCC cells by targeting PTEN via PI3K/Akt pathway. However, the clinical significance of miR‐106b‐5p expression in HCC and its underlying molecular mechanisms remain unclear.

To address this problem, we firstly detected the expression levels of miR‐106b‐5p in 108 HCC clinical samples by quantitative real‐time reverse transcription PCR. Then, the associations of miR‐106b‐5p expression with various clinicopathological features and patients' prognosis were evaluated by Chi‐square test, Kaplan‐Meier, and Cox proportional regression analyses, respectively. In addition, the interaction between miR‐106b‐5p and its target gene was verified by dual luciferase reporter gene system. CCK‐8 assay and Transwell Matrigel invasion assay were used to measure the roles of miR‐106b‐5p and its target gene in HCC cell viability and invasion abilities, respectively.

## 2 MATERIALS AND METHODS

### 2.1 Patients and tissue samples

The current study used the same clinical cohort with our previous study,^22^ and was approved by the Research Ethics Committee of Xinjiang Medical University. Informed consent was obtained from all of the patients. All specimens were handled and made anonymous according to the ethical and legal standards. This clinical cohort consists a total of 108 HCC patients and the detailed information is provided in Supplementary File S1‐section 1.

### 2.2 Cell lines

Two human HCC cell lines HepG2 and SMMC‐7721 were purchased from the cell bank of the Chinese Academy of Sciences. The cells were cultured in DMEM (Invitrogen) supplemented with 10% fetal bovine serum (Invitrogen), 2 mmol/L glutamine, 100 U of penicillin/mL, and 100 µg of streptomycin/ml (Cambrex). The cells were incubated at 37°C in a humidified incubator containing 5% CO_2_.

### 2.3 Quantitative real‐time reverse transcription PCR analysis

Quantitative real‐time reverse transcription PCR was performed to detect the expression of miR‐106b‐5p in HCC clinical samples and cell lines as our previous description.^22^ The detailed information is provided in Supplementary File S1‐section 2. Primers used in this study were as follows according to the previous study by Yang et al^23^ The primer sequences for miR‐106b‐5p: 5′‐TGC GGC AAC ACC AGT CGA TGG‐3′ (forward) and 5′‐CCA GTG CAG GGT CCG AGG T‐3′ (reverse). The primer sequences for U6: 5′‐CTC GCT TCG GGC AGC ACA‐3′ (forward) and 5′‐AAC GCT TCA CGA ATT TGC GT‐3′ (reverse). The primer sequences for RUNX3 mRNA: 5′‐ GAG TTT CAC CCT GAC CAT CAC TGT G‐3′ (forward) and 5′‐ GCC CAT CAC TGG TCT TGA AGG TTG‐3′ (reverse). The primer sequences for β‐actin: 5′‐ TTC CTT CTT GGG TAT GGA AT‐3′ (forward) and 5′‐ GAG CAA TGA TCT TGA TCT TC‐3′ (reverse). Primers were synthesized by Sangon Biotech Shanghai Co. Ltd. (Shanghai, China).

### 2.4 Dual‐Luciferase reporter assay

Two human HCC cell lines HepG2 and SMMC‐7721 (1 × 10^5^/well) were seeded onto a 24‐well plate and co‐transfected with luciferase reporter constructs encoding the wild‐type 3′‐UTR of RUNX3 (RUNX3‐WT‐3′‐UTR) or a mutated 3′‐UTR of RUNX3 (RUNX3‐MUT‐3′‐UTR) (RiboBio) and miR‐106b‐5p mimic (mimic‐106b) or negative control mimic (mimic‐NC) (Sangon Biotech Shanghai Co. Ltd.) using Lipofectamine 2000 (Invitrogen) based on the protocol provided by the manufacturer. The luciferase and renilla signals were measured at 24 hours after the cell transfection using the Dual Luciferase Reporter Assay Kit (Promega) based on the protocol provided by the manufacturer. The relative luciferase activity was normalized to Renilla luciferase activity. Each sample was detected in triplicate.

### 2.5 Cellular transfection

For cellular transfection, 5 × 10^3^ HepG2 and SMMC‐7721 cells were respectively seeded into each well of 96‐well plate and incubated overnight. When cells were grown to 60%‐80% confluence, the transfection of mimic‐106b/mimic‐NC (Sangon Biotech Shanghai Co. Ltd.), miR‐106b‐5p inhibitor vector (anti‐miR‐106b)/anti‐NC (GenePharma), and/or RUNX3 expression vector (RUNX3‐vector)/negative control vector (NC‐vector) (Sangon Biotech Shanghai Co. Ltd.) were performed Lipofectamine 2000 (Invitrogen) based on the manufacturer's instructions.

### 2.6 Western blot analysis

Expression levels of RUNX3 protein in HCC cells transfected with mimic‐106b/mimic‐NC and/or RUNX3‐vector/NC‐vector were measured by western blot analysis. Total protein was extracted from HCC cells and quantified by the Bradford assay (Bio‐Rad). Then, the identical quantities of proteins were separated by sodium dodecyl sulfatepolyacrylamide gel electrophoresis (SDS‐PAGE) and transferred onto PVDF membranes (Bio‐Rad). After incubated with primary antibody of RUNX3 (goat anti‐human RUNX3 affinity purified polyclonal antibody, dilution 1:500, Santa Cruz Biotech) overnight at 37°C, the membranes were then treated with horseradish peroxidase‐conjugated secondary antibody (donkey anti‐goat secondary antibody, dilution 1:1000, Santa Cruz Biotech) for 1 hour at 37°C. GAPDH was used as a internal control. Finally, the Bio‐Rad Gel imaging system (Bio‐Rad) was used to quantify protein expression data.

### 2.7 Cell viability assay

Cell viability of HCC cell lines after transfected with mimic‐106b/mimic‐NC, anti‐miR‐106b/anti‐NC, and/or RUNX3‐vector/NC‐vector was assessed by the 3‐(4,5‐dimethylthiazol‐2‐yl)‐2,5‐diphenyltetrazolium bromide (MTT) assay as our previous description.^22^ The detailed information is provided in Supplementary File S1‐section 3.

### 2.8 Transwell matrigel invasion assay

Cell invasion ability of HCC cell lines after transfected with mimic‐106b/mimic‐NC, anti‐miR‐106b/anti‐NC, and/or RUNX3‐vector/NC‐vector was assessed by the Transwell Matrigel invasion assay as our previous description.^22^ The detailed information is provided in Supplementary File S1‐section 4.

### 2.9 Statistical analysis

Statistical analysis was performed using SPSS11.0 software for Windows (SPSS Inc). Continuous variables were shown as $\bar{X}\pm s$. The Chi‐square test was used to evaluate the associations of miR‐106b‐5p expression with various clinicopathological features of HCC patients. Correlations of miR‐106b‐5p expression with overall and recurrence‐free survivals of HCC patients were estimated by the Kaplan‐Meier method, and the resulting curves were compared using the log‐rank test. Cox proportional hazard models were used to identify the independent prognostic factors for overall and recurrence‐free survivals of HCC patients. Student's *t* test or one‐way ANOVA was used for the comparisons between groups. A *P*‐value of less than 0.05 was considered statistically significant.

## 3 RESULTS

### 3.1 Increased expression of miR‐106b‐5p associates with aggressive progression of clinical HCC samples

**Figure 1:**
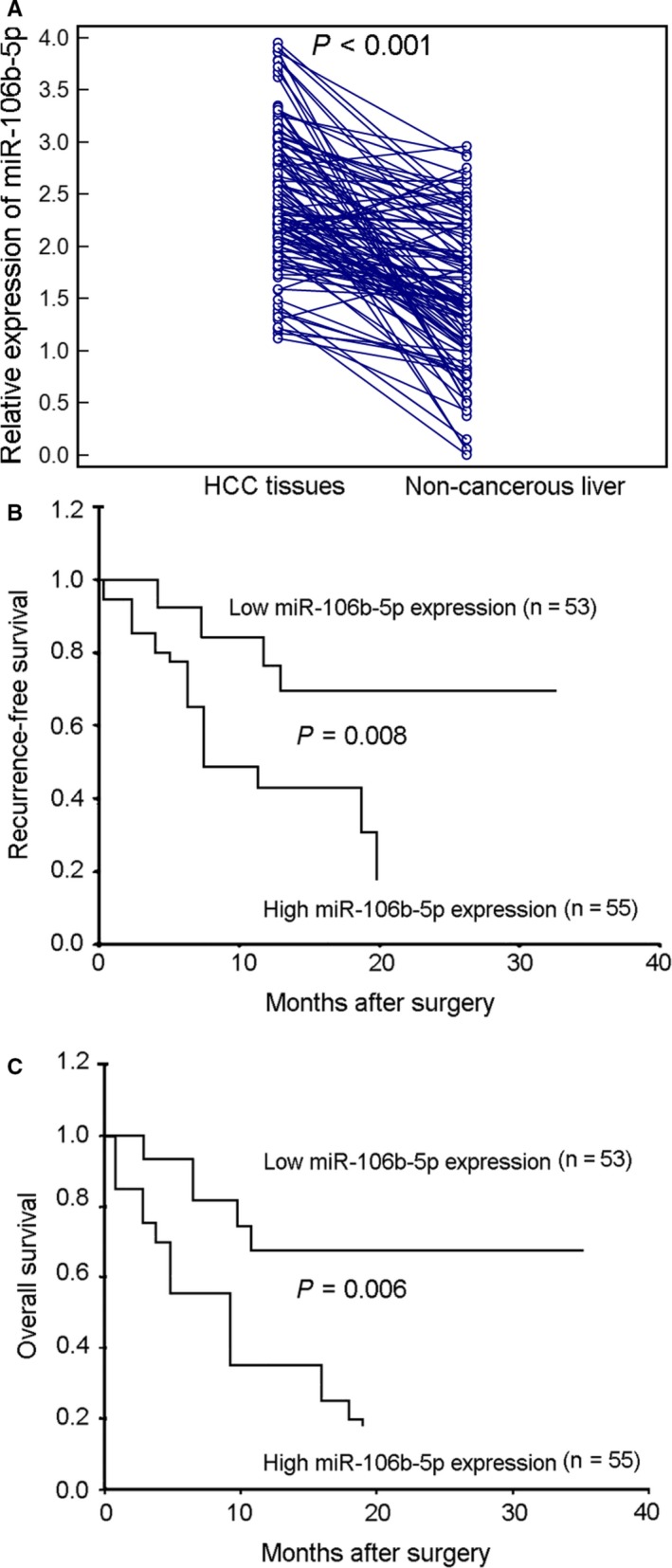
Expression patterns and prognostic value of miR‐106b‐5p in HCC. A, The relative expression levels of miR‐106b‐5p were significantly higher in HCC tissues compared with paired noncancerous adjacent liver tissues measured by quantitative real‐time reverse transcription PCR analysis (*P* < .001). B, Kaplan‐Meier survival curves for recurrence‐free survival are plotted according to miR‐106b‐5p expression (compared with log‐rank test). C, Kaplan‐Meier survival curves for overall survival are plotted according to miR‐106b‐5p expression (compared with log‐rank test)

The expression levels of miR‐106b‐5p in HCC tissues were significantly higher than that in noncancerous adjacent liver tissues (HCC vs Noncancerous liver: 2.46 ± 0.67 vs 1.56 ± 0.69, *P* < .001, Figure 1A). All 108 HCC patients were divided into two groups using the median value of miR‐106b‐5p levels as a cutoff (2.35, normalized to RNU6B): high miR‐106b‐5p expression group (n = 55, 3.00 ± 0.45) and low miR‐106b‐5p expression group (n = 53, 1.92 ± 0.35). As shown in Table 1, HCC patients with advanced TNM stage more frequently displayed high miR‐106b‐5p expression than those with early TNM stage (*P* = .01). No significant differences were found in age, sex, serum AFP level, tumor size, tumor number, histological grade, cirrhosis status, and hepatitis B infection status between high miR‐106b‐5p expression group and low miR‐106b‐5p expression group (all *P* > .05, Table 1).

**Table 1:** Associations between miR‐106b‐5p expression and various clinicopathological features in 108 HCC patients

Features	NO.	miR‐106b‐5p expression (n, %)		*P*
		Low	High	
Gender				
Male	63	28 (44.44)	35 (55.56)	.08
Female	45	25 (55.56)	20 (44.44)	
Age at diagnosis				
≥50	60	31 (51.67)	29 (48.33)	.09
<50	48	22 (45.83)	26 (54.17)	
Serum AFP level (ng/mL)				
≥25	63	28 (44.44)	35 (55.56)	.08
<25	45	25 (55.56)	20 (44.44)	
Tumor size (cm)				
≥5	58	23 (39.66)	35 (60.34)	.05
<5	50	30 (60.00)	20 (40.00)	
Tumor number				
Solitary	42	19 (45.24)	23 (54.76)	.26
Multiple	66	34 (51.52)	32 (48.48)	
TNM stage				
I	18	15 (83.33)	3 (16.67)	.02
II	30	20 (66.67)	10 (33.33)	
III	38	18 (47.37)	20 (52.63)	
IV	22	0 (0)	22 (100.00)	
Histological grade				
High	26	8 (30.77)	18 (69.23)	.05
Low	82	45 (54.88)	37 (45.12)	
Cirrhosis				
Negative	48	23 (47.92)	25 (52.08)	.09
Positive	60	30 (50.00)	30 (50.00)	
Hepatitis B				
Negative	16	11 (68.75)	5 (31.25)	.06
Positive	92	42 (45.65)	50 (54.35)	

### 3.2 Increased expression of miR‐106b‐5p associates with poor prognosis of HCC patients

The detailed clinical information of all 108 HCC patients was reviewed to evaluate the prognostic impact of miR‐106b‐5p expression in HCC patients. Kaplan‐Meier curve analysis and log‐rank test demonstrated that HCC patients with high miR‐106b‐5p expression had shorter recurrence‐free and overall survivals than those with low miR‐106b‐5p expression (for recurrence‐free survival and overall survival: log‐rank values 6.896 and 7.286, *P* = .008 and 0.006, respectively. Figure 1B,C, Table 2). Moreover, multivariate Cox regression analysis enrolling the significant clinicopathological parameters based on the univariate analysis identified miR‐106b‐5p expression as an independent prognostic factor for recurrence‐free survival and overall survival of HCC patients (for recurrence‐free survival: Hazard ratio [HR] 5.101, *P* = .02; for overall survival: HR 7.268, *P* = .01, Table 3).

**Table 2:** Univariate analysis on the associations of prognosis with various clinicopathologic parameters and miR‐106b‐5p expression in HCC patients

Features	Recurrence‐free survival			Overall survival		
	HR	95% CI	*P*	HR	95% CI	*P*
Gender	0.712	0.328‐1.296	.3	1.007	0.426‐2.335	.8
Age at diagnosis	0.669	0.358‐1.402	.2	0.890	0.368‐1.563	.4
Histological grade	3.981	0.665‐8.879	.03	4.107	0.719‐9.989	.02
TNM stage	6.837	1.758‐20.376	.008	6.502	1.269‐19.668	.008
Tumor size	1.405	1.959‐5.027	.1	1.386	1.893‐4.165	.1
Serum AFP level	2.700	1.317‐5.543	.1	1.613	0.700‐4.253	.2
Tumor number	2.876	1.790‐5.533	.1	1.508	1.431‐4.383	.2
Cirrhosis	2.211	1.087‐7.269	.1	2.468	1.507‐6.607	.1
Hepatitis B	1.903	1.212‐4.954	.2	1.782	1.379‐4.186	.2
miR‐106b‐5p expression	6.896	1.682‐20.932	.008	7.286	1.879‐22.668	.006

**Table 3:** Multivariate analysis on the associations of prognosis with various clinicopathologic parameters and miR‐106b‐5p expression in HCC patients

Features	Recurrence‐free survival			Overall survival		
	HR	95% CI	*P*	HR	95% CI	*P*
Histological grade	0.218	0.008‐7.091	.4	1.662	0.108‐26.089	.8
TNM stage	5.103	1.366‐13.860	.02	7.326	1.103‐21.916	.01
MiR‐106b‐5p expression	5.101	1.328‐12.968	.02	7.268	1.122‐21.260	.01

### 3.3 Increased expression of miR‐106b‐5p promotes cell viability and invasion of human HCC cells

**Figure 2:**
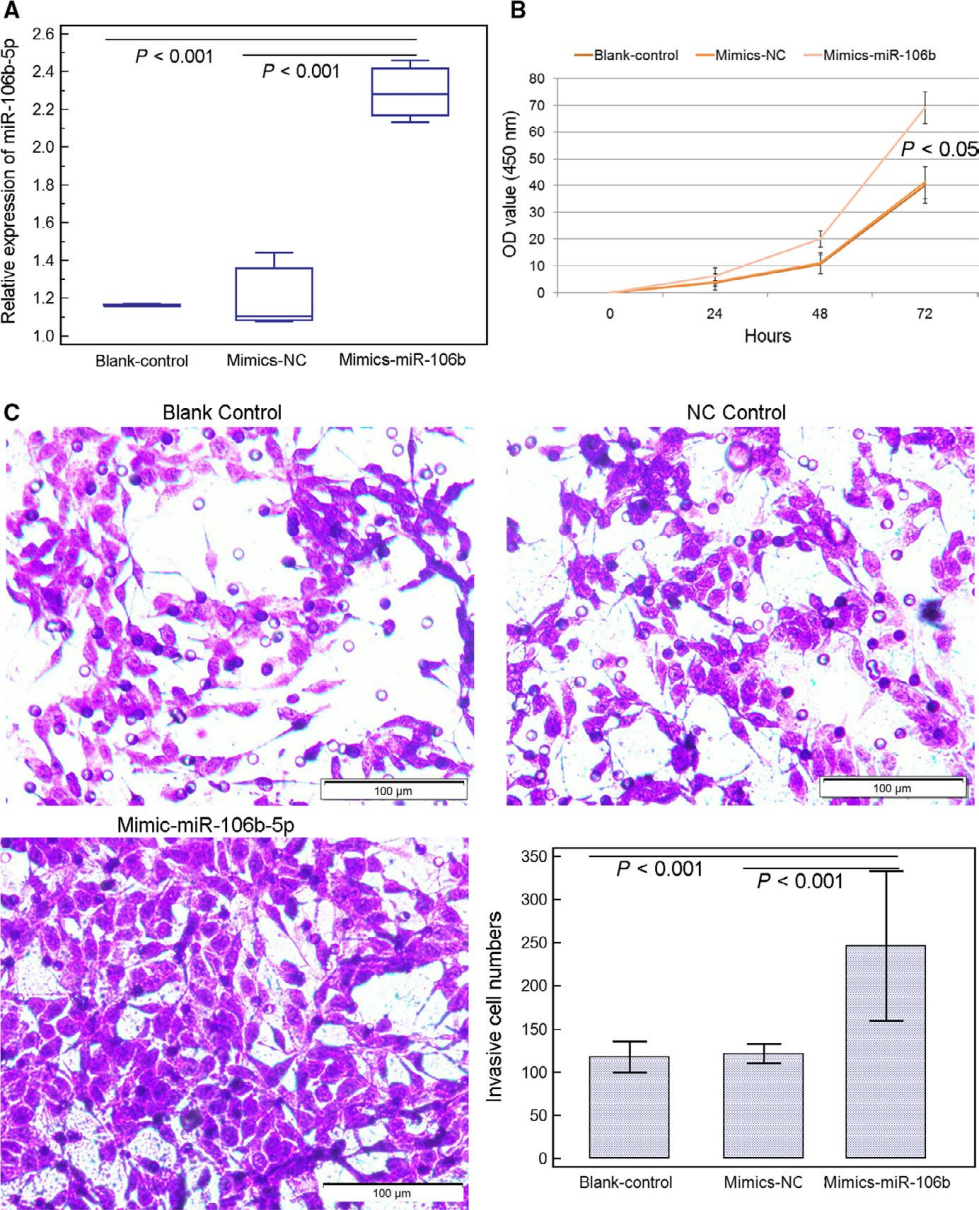
MiR‐106b‐5p promotes cell viability and invasion of SMMC7721 cell line. A, Expression levels of miR‐106b‐5p in SMMC7721 cells transfected with mimic‐miR‐106b‐5p or mimic‐NC was detected by quantitative real‐time reverse transcription PCR. The results are the mean ± SD of triplicate experiments. B, miR‐106b‐5p overexpression significantly enhanced the viability of SMMC7721 cells (*P* < .05). C, miR‐106b‐5p overexpression significantly promoted the invasion ability of SMMC7721 cells (*P* < .001). The results are the mean ± SD of triplicate experiments

**Figure 3:**
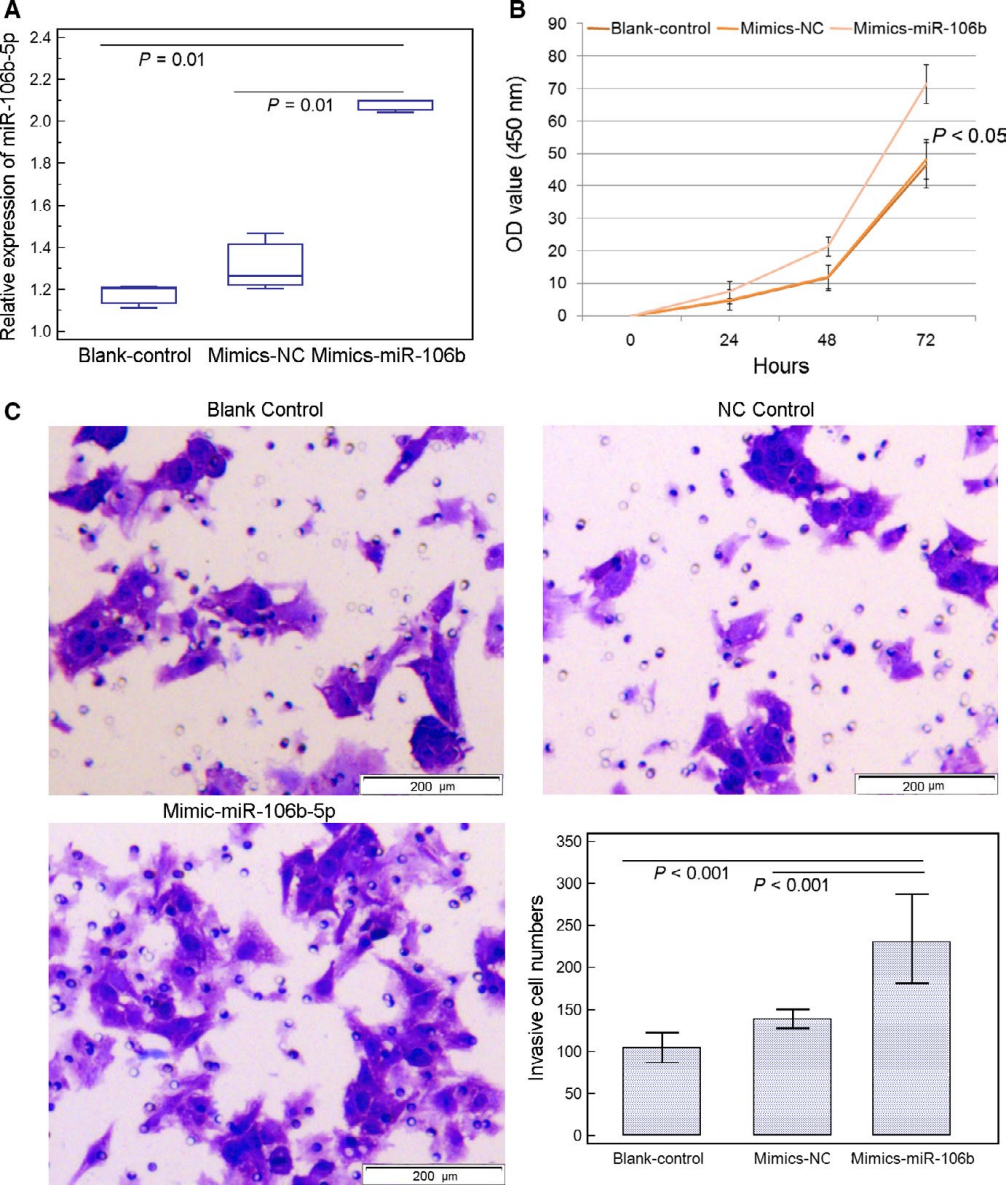
Increased expression of miR‐106b‐5p promotes cell viability and invasion of HepG2 cell line. A, Expression levels of miR‐106b‐5p in HepG2 cells transfected with mimic‐miR‐106b‐5p or mimic‐NC was detected by quantitative real‐time reverse transcription PCR. The results are the mean ± SD of triplicate experiments. B, miR‐106b‐5p overexpression significantly enhanced the viability of HepG2 cells (*P* < .05). C, miR‐106b‐5p overexpression significantly promoted the invasion ability of HepG2 cells (*P* < .001). The results are the mean ± SD of triplicate experiments

The above evidence show the significant associations of miR‐106b‐5p upregulation with aggressive progression and poor prognosis in HCC patients and prompt us to determine the oncogenic functions of miR‐106b‐5p in human HCC cell lines in vitro. As shown in Figures 2A and 3A, the expression levels of miR‐106b‐5p in SMMC7721 and HepG2 cells transfected with miR‐106b‐5p‐mimics were dramatically elevated compared with cells transfected with negative control‐mimics (for SMMC7721 cells: *P* < .001; for HepG2 cells: *P* = .02). Functionally, the enforced expression of miR‐106b‐5p significantly promoted the proliferative activity of both SMMC7721 and HepG2 cells (both *P* = .01, Figures 2B and 3B). In addition, the over‐expression of miR‐106b‐5p led to a significant increase in cell invasion of SMMC7721 and HepG2 cells (*P* = .008, Figures 2C and 3C).

### 3.4 Reduced expression of miR‐106b‐5p suppresses cell viability and invasion of human HCC cells

**Figure 4:**
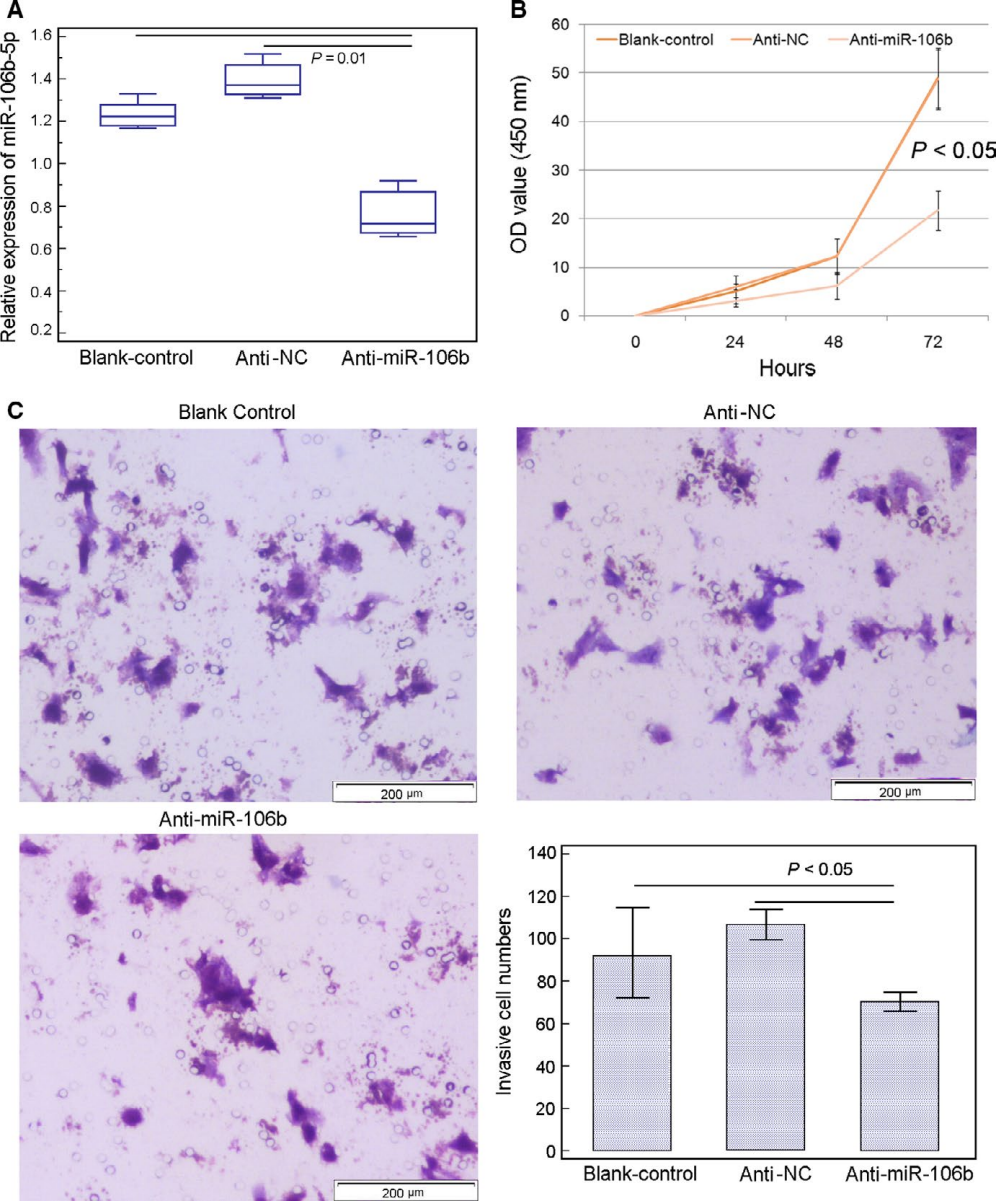
Reduced expression of miR‐106b‐5p suppresses cell viability and invasion of HepG2 cell line. A, Expression levels of miR‐106b‐5p in HepG2 cells transfected with anti‐miR‐106b‐5p or anti‐NC was detected by quantitative real‐time reverse transcription PCR. The results are the mean ± SD of triplicate experiments. B, Reduced expression of miR‐106b‐5p significantly suppressed the viability of HepG2 cells (*P* < .05). C, Reduced expression of miR‐106b‐5p significantly suppressed the invasion ability of HepG2 cells (*P* < .05). The results are the mean ± SD of triplicate experiments

Following the transfection with miR‐106b‐5p inhibitor vector (anti‐miR‐106b), the expression levels of miR‐106b‐5p in HepG2 cells were significantly reduced, when compared with those transfected with anti‐NC (*P* = .01, Figure 4A). Functionally, the reduced expression of miR‐106b‐5p markedly suppressed the proliferative (*P* = .03, Figure 4B) and invasive (*P* = .03, Figure 4C) activities of HepG2 cells.

### 3.5 RUNX3 acts as a direct target of miR‐106b‐5p in human HCC cells

**Figure 5:**
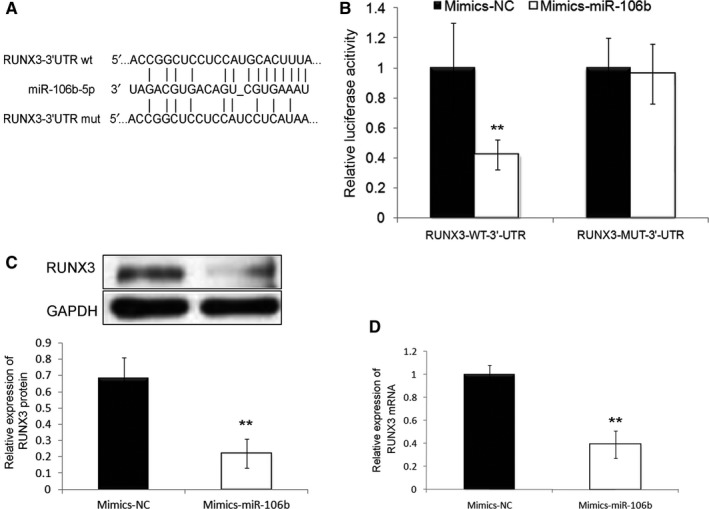
RUNX3 was a direct target gene of miR‐106b‐5p in SMMC7721 cell line. A, A putative binding site for miR‐106b‐5p identified in the 3'‐UTR of RUNX3. B, The enforced expression of miR‐106b‐5p decreased the luciferase activity associated with the RUNX3‐WT‐3'‐UTR (**P < .01) but did not affect that with the RUNX3‐MUT‐3'‐UTR. C, Western blot analysis demonstrated that the expression level of RUNX3 protein was significantly suppressed in the SMMC7721 cells overexpressed miR‐106b‐5p relative to those transfected with miR‐NC (**P < .01). D, Quantitative real‐time reverse transcription PCR analysis demonstrated that the expression level of RUNX3 mRNA was significantly suppressed in the SMMC7721 cells overexpressed miR‐106b‐5p relative to those transfected with miR‐NC (**P < .01)

**Figure 6:**
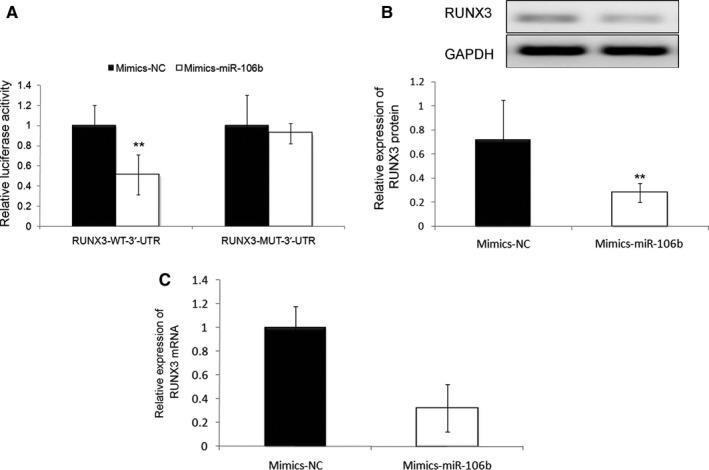
RUNX3 was a direct target gene of miR‐106b‐5p in HepG2 cell line. A, The enforced expression of miR‐106b‐5p decreased the luciferase activity associated with the RUNX3‐WT‐3'‐UTR (**P < .01) but did not affect that with the RUNX3‐MUT‐3'‐UTR. B, Western blot analysis demonstrated that the expression level of RUNX3 protein was significantly suppressed in the HepG2 cells overexpressed miR‐106b‐5p relative to those transfected with miR‐NC (**P < .01). C, Quantitative real‐time reverse transcription PCR analysis demonstrated that the expression level of RUNX3 mRNA was significantly suppressed in the HepG2 cells overexpressed miR‐106b‐5p relative to those transfected with miR‐NC (**P < .01)

To elucidate the underlying molecular mechanisms of miR‐106b‐5p involved in HCC, we collected the potential target genes of miR‐106b‐5p from the experimentally validated microRNA‐target interactions database (miRTarBase, http://mirtarbase.mbc.nctu.edu.tw/php/index.php, Release 7.0: September 15, 2017). We only chose the interactions between miR‐106b‐5p and potential targets which were validated using Luciferase reporter assay. As a result, *runt‐related transcription factor 3 (RUNX3)*, a well‐known tumor suppressor in HCC, was predicted to be a hypothetic target gene of miR‐106b‐5p in this malignancy. Then, a putative binding site for miR‐106b‐5p was identified in the 3'‐UTR of RUNX3, and luciferase reporter assay was carried out in HCC cells co‐transfected with miR‐106b‐5p mimics or miR‐NC and RUNX3‐WT‐3′‐UTR or RUNX3‐MUT‐3′‐UTR (Figure 5A). As shown in Figures 5B and 6A, the enforced expression of miR‐106b‐5p decreased the luciferase activity associated with the RUNX3‐WT‐3′‐UTR (both *P* < .01), but did not affect that with the RUNX3‐MUT‐3′‐UTR based on both SMMC7721 and HepG2 cells. In addition, western blot analysis demonstrated that the expression levels of RUNX3 protein were significantly suppressed in both SMMC7721 and HepG2 cells which were transfected with mimic‐miR‐106b‐5p relative to those transfected with miR‐NC (both *P* < .01, Figures 5C and 6B), which was consistent with the findings of the quantitative real‐time reverse transcription PCR analysis (both *P* < .01, Figures 5D and 6C).

The above data suggest that RUNX3 may a direct target gene of miR‐106b‐5p in HCC.

### 3.6 RUNX3 over‐expression reverses the oncogenic effects of miR‐106b‐5p

**Figure 7:**
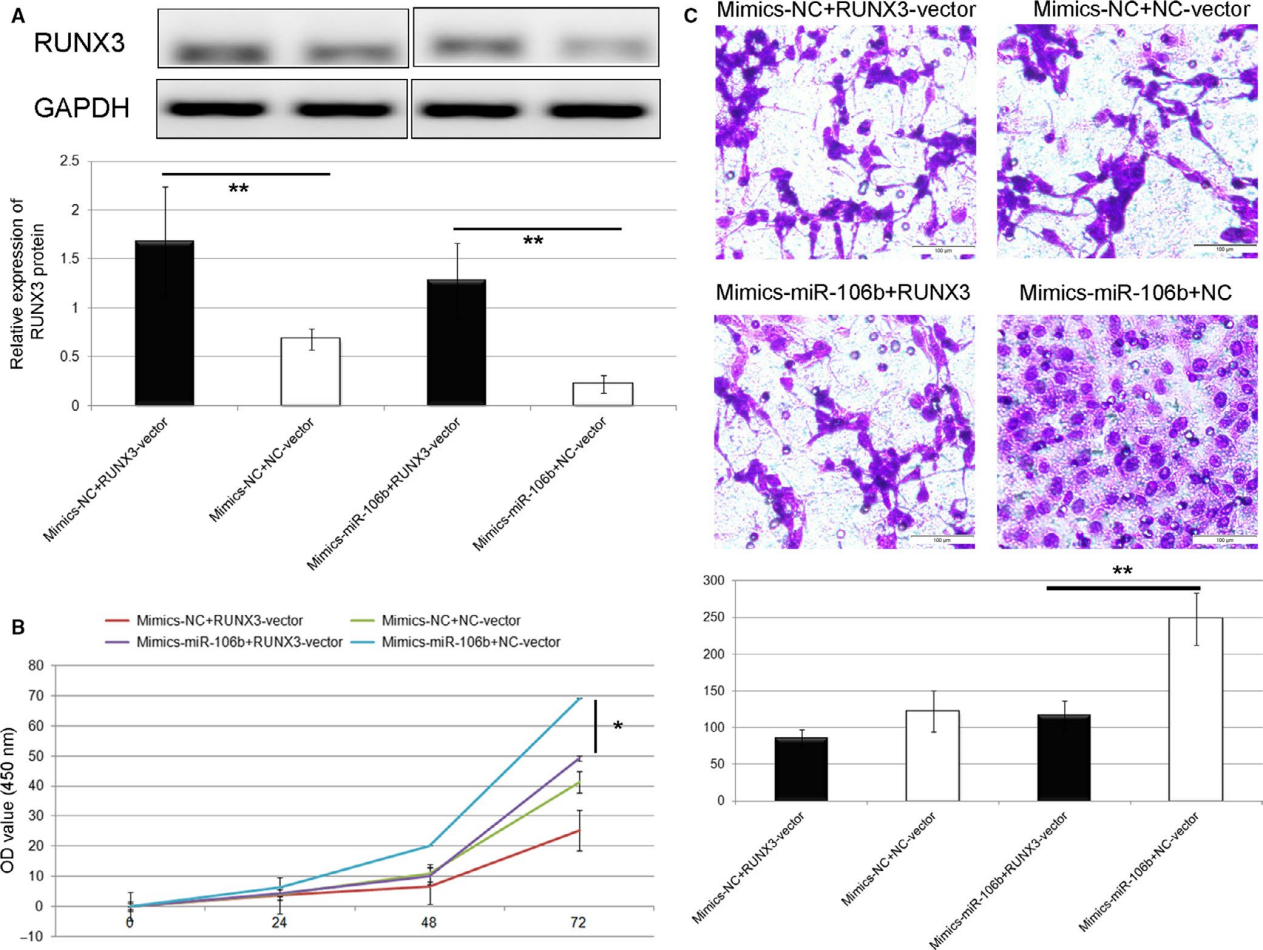
RUNX3 over‐expression reverses the oncogenic effects of miR‐106b‐5p in SMMC7721 cell line. A, Western blot analysis confirmed that the downregulation of RUNX3 protein expression caused by the upregulation of miR‐106b‐5p was recovered by the co‐transfection with RUNX3‐vector in HSMMC7721 cells (** *P* < .01). B and C, The restored RUNX3 expression significantly rescued the effects of exogenous miR‐106b‐5p on the viability (* *P* < .05) and invasion (** *P* < .01) of SMMC7721 cells

**Figure 8:**
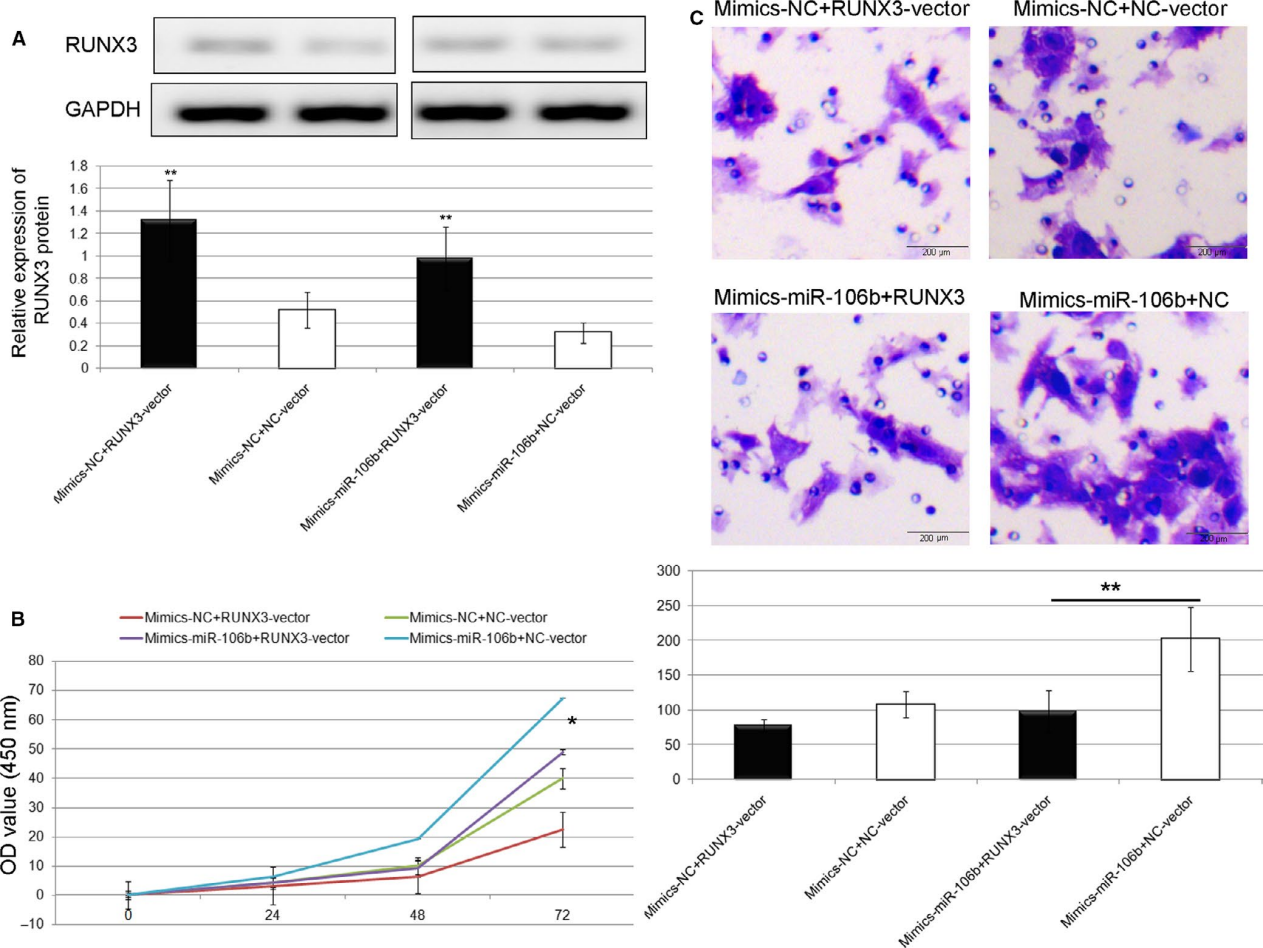
RUNX3 over‐expression reverses the oncogenic effects of miR‐106b‐5p in HepG2 cell line. A, Western blot analysis confirmed that the downregulation of RUNX3 protein expression caused by the upregulation of miR‐106b‐5p was recovered by the co‐transfection with RUNX3‐vector in HepG2 cells (** *P* < .01). B and C, The restored RUNX3 expression significantly rescued the effects of exogenous miR‐106b‐5p on the viability (* *P* < .05) and invasion (** *P* < .01) of HepG2 cells

To verify whether the oncogenic roles of miR‐106b‐5p in HCC are mediated by RUNX3, we performed a series of rescue experiments by co‐transfection of miR‐106b‐5p mimics in SMMC7721 cells together with RUNX3‐vector or NC‐vector. Western blot analysis confirmed that the downregulation of RUNX3 protein expression caused by the upregulation of miR‐106b‐5p was recovered by the co‐transfection with RUNX3‐vector in SMMC7721 cells (Figure 7A, *P* < .01). Moreover, the restored RUNX3 expression significantly rescued the effects of exogenous miR‐106b‐5p on the viability (Figure 7B, *P* < .05) and invasion (Figure 6C, *P* < .01) of SMMC7721 cells. The similar findings were also verified using HepG2 cells as shown in Figure 8.

## 4 DISCUSSION

Understanding the molecular mechanisms involved into HCC carcinogenesis and progression may be critical to identify novel therapeutic targets and improve the clinical outcome of HCC patients. In this study, we revealed the overexpression of miR‐106b‐5p in HCC tissues, which was dramatically associated with advanced TNM stage, short recurrence‐free and overall survivals. Importantly, miR‐106b‐5p expression was an independent predictor of poor prognosis in patients with HCC. To elucidate the regulatory mechanism of miR‐106b‐5p, we identified RUNX3 as its direct target gene in HCC cells. Functionally, miR‐106b‐5p upregulation promoted the viability and invasion of HCC cells, while enforced RUNX3 expression reversed the oncogenic effects of miR‐106b‐5p overexpression. These findings confirm that miR‐106b‐5p may exert its oncogenic roles in HCC, at least in part, by inhibiting RUNX3.

Growing evidence show that miRNAs may be potential therapeutic targets for their roles in regulating either oncogenes or tumor suppressor genes. Recent studies have revealed that miR‐106b‐5p may play an oncogenic role in various cancer types by promoting malignant cell viability and invasion.^14^, ^15^, ^16^, ^17^, ^18^, ^19^, ^20^ For example, Lu et al^14^ revealed that miR‐106b‐5p could mediate the constitutive activation of Wnt/β‐catenin signaling, and promote renal cell carcinoma aggressiveness and stem cell‐like phenotypes; Wei et al^15^ indicated that miR‐106b‐5p overexpression promoted proliferation and inhibited apoptosis by downregulating BTG3 expression in vitro, as well as xenograft tumor formation in vivo; Liu et al^16^ found that miR‐106b‐5p could boost glioma tumorigensis by targeting multiple tumor suppressor genes, including RBL1, RBL2, and CASP8; Yu et al^17^ demonstrated that miR‐106b‐5p enhanced the sensitivity of nonsmall‐cell lung cancer cell line A549/DDP to cisplatin by targeting the expression of PKD2. Consistent to these previous studies, our data here confirmed the upregulation of miR‐106b‐5p in HCC tissues compared with noncancerous adjacent liver tissues, and also demonstrated the significant associations of miR‐106b‐5p overexpression with advanced TNM stage, as well as short recurrence‐free and overall survivals. Notably, the oncogenic roles of miR‐106b‐5p in promoting cell proliferative and invasive abilities of HCC cells were further verified in vitro.

miRNAs exert the corresponding functions via regulating their target genes. To investigate the molecular mechanisms underlying the oncogenic roles of miR‐106b‐5p in HCC, we identified the potential target gene of this miRNA by luciferase reporter assay and western blot analysis. RUNX3 was identified as one of direct target genes for miR‐106b‐5p in HCC cells. RUNX3 function as an important transcription factor of the TGF‐β‐mediated signaling pathway. It plays a tumor suppressive role in various human cancers, including oral squamous cell carcinoma, laryngeal carcinoma, breast cancer, lung cancer, HCC, gastric cancer, pancreatic cancer, and colorectal cancer.^24^, ^25^, ^26^, ^27^, ^28^, ^29^, ^30^ Notably, Yang et al^23^ indicated that the upregulation of RUNX3 after miR‐106b‐5p suppression implying that RUNX3 might be a tumor‐suppressor in retinoblastoma and a target of miR‐106b‐5p. Xu et al^30^ also reported that the loss of RUNX3 expression was correlated with the upregulation of miR‐106b‐5p in human laryngeal carcinoma tissues, and miR‐106b promoted the viability and invasion of laryngeal carcinoma cells by directly targeting RUNX3. Similarly, our data showed that the downregulation of RUNX3 might be mediated by miR‐106b through binding its 3′‐UTR. Moreover, we observed that miR‐106b promoted the viability and invasion of HCC cells by directly targeting RUNX3, and RUXN3 knockdown might abolish this phenotype.

In conclusion, our data offer the convincing evidence that miR‐106b‐5p may serve as a potent prognostic marker for tumor recurrence and survival of HCC patients. miR‐106b‐5p may exert an oncogenic role in HCC via regulating its target gene RUNX3.

## CONFLICT OF INTEREST

None.

## AUTHOR CONTRIBUTIONS

All authors participated in the design, interpretation of the studies and analysis of the data and review of the manuscript. GH, GS, ZX, and ZS conducted the experiments. ZD, LJ, and HS collected clinical samples. GH and HW wrote the manuscript and performed data analysis.
