# Supplementary material for: miR‐106b‐5p promotes aggressive progression of hepatocellular carcinoma via targeting RUNX3
Source: Cancer Med. 2019 Sep 10;8(15):6756–67. doi: 10.1002/cam4.2511 (PMC6825988; doi:10.1002/cam4.2511)
Supplement: Supplementary file 1 [file CAM4-8-6756-s001.docx]

**Supplementary File S1 for " Overexpression of microRNA-106b-5p confers poor prognosis and promotes proliferation as well as invasion in hepatocellular carcinoma via suppressing Runt-related transcription factor 3"**

*Section 1: Patients and Tissue Samples*

The study was approved by the Research Ethics Committee of Xinjiang Medical University, 302 Hospital of PLA and 301 Hospital of PLA, P.R.China. Informed consent was obtained from all of the patients. All specimens were handled and made anonymous according to the ethical and legal standards.

We retrospectively investigated 108 patients (63 men and 45 women) with HCC who underwent routine curative surgery between April 2001 and March 2009 at Xinjiang Medical University, 302 Hospital of PLA and 301 Hospital of PLA. None of the patients received radiotherapy or chemotherapy before surgery. All patients included in the study had been newly diagnosed with HCC. For each case, the diagnosis and the histological grade were confirmed by two pathologists. The fresh human HCC tissues and paired normal adjacent tissues were obtained from each HCC patients. Tissues were snap-frozen in liquid nitrogen after surgical resection until use.

One-hundred and eight HCC patients were received follow-up (range: 6~38 months; median: 16 months). Up to February 2012, 58 (53.7%) patients were alive and 50 (46.3%) patients had died of progression of this disease. Follow-up usually included serum α-fetoprotein (AFP) level, abdominal ultrasonography, and chest radiography every 1-3 months after curative hepatectomy. When tumor recurrence was suspected, computed tomography scan or/and magnetic resonance imaging scan was performed to confirm the diagnosis. Overall survival was calculated as the interval between the date of surgery and either the date of death or the last follow-up date of the patient. Recurrence-free survival was calculated as the time from the date of surgery to the date of tumor recurrence and was censored at the time of last follow-up or death if there was no evidence of tumor recurrence at that time.

*Section 2: Quantitative real-time reverse transcription PCR analysis*

Total RNA was extracted from liver tissues or cells using TRIzol reagent (Invitrogen Corp., Carlsbad, CA, USA), and small RNA isolation and enrichment were performed using the mirVanaTM miRNA Isolation Kit (Applied Biosystems/Ambion Inc., Austin, TX, USA) according to the manufacturer's instructions. RNA concentration and purity were measured using the NanoDrop ND-1000 spectrophotometer (NanoDrop Technologies, Houston, TX, USA). Only the samples with the OD A260/A280 ratio close to value of 2.0, which indicates that the RNA is pure, were subsequently analyzed. cDNA was synthesized from 10 ng of total RNA using TaqManTM MicroRNA hsa-miR-372 specific primer (Applied Biosystems) and a TaqManTM MicroRNA Reverse Transcription Kit (Applied Biosystems). Reverse transcription was performed as described in the previous study [1]. Expression level of miR-106b-5p was normalized to that of the small nuclear RNA RNU6B transcript. Each sample was analyzed in triplicate.

*Section 3: Cell proliferation assay*

Cell proliferation ability of HCC cell lines after transfected with mimic-106b/mimic-NC and/or RUNX3-vector/NC-vector was assessed by the 3-(4,5-dimethylthiazol-2-yl)-2,5-diphenyltetrazolium bromide (MTT) assay. Briefly, 48 h after transfection, 20 μl MTT solution (Sigma, USA) was added into the culture medium for 4 h incubation. Then, 150 µl DMSO (Sigma, USA) was added into each well to dissolve the crystals. The absorbance of each sample was recorded at 490 nm after 10 min for three times.

*Section 4: Transwell Matrigel invasion assay*

Cell invasion ability of HCC cell lines after transfected with mimic-106b/mimic-NC and/or RUNX3-vector/NC-vector was assessed by the Transwell Matrigel invasion assay. Briefly, A 200 μl of the cell suspension (4×10^4^ cells) was added into each well of the upper chamber and the medium containing 10% serum as a chemo-attractant was added into the bottom wells of the 24-well chamber. After the cells were incubated for 20 h at 37°C in a humidified incubator with 5% CO_2_, the non-invading cells that remained on the upper surface of the membrane were removed by scrubbing with a cotton swab. The invasive cells attached to the lower surface of the membrane were fixed in 10% formalin at room temperature for 30 min and stained with 0.05% crystal violet. The number of invasive cells was counted at 200× magnification from ten different fields of each filter. Each experiment was repeated three times.

**References**

1. Yamashita S, Yamamoto H, Mimori K, et al. MicroRNA-372 Is Associated with Poor Prognosis in Colorectal Cancer. Oncology. 2012; 82: 205-212.
